# Proteomic and Phosphoproteomic Insights into a Signaling Hub Role for Cdc14 in Asexual Development and Multiple Stress Responses in *Beauveria bassiana*

**DOI:** 10.1371/journal.pone.0153007

**Published:** 2016-04-07

**Authors:** Zhi-Kang Wang, Jie Wang, Jing Liu, Sheng-Hua Ying, Xiao-Jun Peng, Ming-Guang Feng

**Affiliations:** 1 Institute of Microbiology, College of Life Sciences, Zhejiang University, Hangzhou, 310058, China; 2 Jingjie PTM Biolabs (Hangzhou) Co., Ltd., Hangzhou, 310018, China; Seoul National University, REPUBLIC OF KOREA

## Abstract

Cdc14 is a dual-specificity phosphatase that regulates nuclear behavior by dephosphorylating phosphotyrosine and phosphoserine/phosphothreonine in fungi. Previously, Cdc14 was shown to act as a positive regulator of cytokinesis, asexual development and multiple stress responses in *Beauveria bassiana*, a fungal insect pathogen. This study seeks to gain deep insight into a pivotal role of Cdc14 in the signaling network of *B*. *bassiana* by analyzing the Cdc14-specific proteome and phosphoproteome generated by the 8-plex iTRAQ labeling and MS/MS analysis of peptides and phosphopeptides. Under normal conditions, 154 proteins and 86 phosphorylation sites in 67 phosphoproteins were upregulated in Δ*cdc14* versus wild-type, whereas 117 proteins and 85 phosphorylation sites in 58 phosphoproteins were significantly downregulated. Co-cultivation of Δ*cdc14* with NaCl (1 M), H_2_O_2_ (3 mM) and Congo red (0.15 mg/ml) resulted in the upregulation / downregulation of 23/63, 41/39 and 79/79 proteins and of 127/112, 52/47 and 105/226 phosphorylation sites in 85/92, 45/36 and 79/146 phosphoproteins, respectively. Bioinformatic analyses revealed that Cdc14 could participate in many biological and cellular processes, such as carbohydrate metabolism, glycerophospholipid metabolism, the MAP Kinase signaling pathway, and DNA conformation, by regulating protein expression and key kinase phosphorylation in response to different environmental cues. These indicate that in *B*. *bassiana*, Cdc14 is a vital regulator of not only protein expression but also many phosphorylation events involved in developmental and stress-responsive pathways. Fourteen conserved and novel motifs were identified in the fungal phosphorylation events.

## Introduction

Eukaryotic development requires a fine coordination of nuclear division with growth and the cell cycle [1]. The nuclear behavior is regulated by Cdc14, a key dual-specificity phosphatase that is highly conserved in eukaryotes and can dephosphorylate the residues of phosphotyrosine and phosphoserine/phosphothreonine [2]. In *Saccharomyces cerevisiae*, Cdc14 can inactivate cyclin dependent kinases (CDKs) at the end of mitosis for cell entry into G1 phase [3, 4] and act as a hub of five phosphatases and 23 kinases [5], including mitosis-associated CDKs and mitogen-activated protein (MAP) kinase cascades involved in cellular stress responses. Although most previous studies in yeast focused primarily on the Cdc14 contribution to nuclear and developmental events, the revealed interaction of Cdc14 with the stress-responsive high-osmolarity glycerol (HOG) and cell wall integrity (CWI) pathways [5] suggests a tight link of this phosphatase to multiple stress responses. This speculation has been experimentally confirmed in *Beauveria bassiana*, a filamentous fungal insect pathogen. Deletion of *cdc14* in *B*. *bassiana* resulted in suppressed transcript profiles of numerous signaling and effector genes involved in developmental and stress-responsive pathways [6]. The *B*. *bassiana* Cdc14 is characteristic of a highly conserved signature motif typical for the superfamily of protein tyrosine phosphatases and four CDK consensus phosphorylation sites (S/TPXK/R), i.e., S_43_PRK_46_, T_414_RIR_417_, S_534_PMR_537_ and S_599_PLR_602_ [6]. Two to six similar sites are also present in other fungal Cdc14 orthologues but none of them exists in *S*. *cerevisiae* [7]. However, a link for Cdc14 to posttranscription and phosphorylation events remains unclear although the transcriptional profiling of many selected genes revealed its hub role in the signaling network of the fungal insect pathogen.

The labeling of isobaric tags for absolute and relative quantification (iTRAQ) is a reliable strategy for quantitative omics-scale [8–10] and has been widely applied for the characterization of proteomes and phosphoproteomes in filamentous fungi, such as *Aspergilli* [11–14], *Fusarium graminearum* [15], *Neurospora crassa* [16], *Phanerochaete chrysosporium* [17] and *Trichoderma reesei* [18]. An advantage of the iTRAQ labeling over other chemical labeling strategies lies in the feasibility for simultaneous analysis of up to eight biological samples in one experiment by labeling peptides (primary amino groups) with isobaric tags that differ in reporter and balancer groups [19–22]. Based on the fully MS/MS-dependent labeling technology, quantitative information on sequenced (fragmented) peptides can be collected by comparing unique reporter groups in the fragmentation spectrum. Protein abundance can be estimated as the number of sampled events of the fragmented peptides.

Fungal insect pathogens used for the development of fungal insecticides rely upon desirable conidiation capacity, virulence and multiple stress tolerance, which are determinants for their potential against arthropod pests [23, 24]. A large number of signaling and effector genes in the developmental and stress-responsive pathways suppressed by the deletion of *cdc14* in *B*. *bassiana* [6] are likely linked to the corresponding proteins and phosphoproteins targeted by Cdc14. Unveiling this link will deepen an insight into the pivotal role of Cdc14 in the fungal infection of host insects and adaptation to diverse host habitats. Therefore, this study seeks to probe protein abundance and phosphorylation events by analyzing the Δ*cdc14*-specific proteome and phosphoproteome generated with the 8-plex iTRAQ labeling strategy. We hope these analyses will reveal the profiles of proteins and phosphoproteins regulated by Cdc14 and hence help to understand the hub role of Cdc14 in the fungal signaling network responsible for asexual development and multiple stress responses.

## Materials and Methods

### Phenotypic Experiments

The wild-type strain *B*. *bassiana* ARSEF 2860 (designated WT) and the Δ*cdc14* and Δ*cdc14/cde14* mutants created in a previous study [6] were grown at an optimal temperature of 25°C in SDAY [Sabouraud dextrose agar (4% glucose, 1% peptone and 1.5% agar) plus 1% yeast extract] for normal growth and conidiation, in SDB (Sabouraud dextrose broth, i.e., agar-free SDAY) for submerged blastospore production, or in MM (minimal medium: 3% sucrose, 0.3% NaNO_3_, 0.1% K_2_HPO_4_, 0.05% KCl, 0.05% MgSO_4_ and 0.001% FeSO_4_ plus 1.5% agar) with altered carbon (glucose, galactose, glycerol or acetate) or nitrogen (NH_4_Cl or NaNO_2_) source for colony growth. Blastospores from 3-day-old SDB cultures were stained with both the nuclear dye DAPI (4′,6′-diamidine-2′-phenylindole dihydrochloride; Sigma, St. Louis, MO, USA) and the cell wall-specific dye Calcofluor white (Sigma) to quantify the number of nuclei in each cell under a confocal microscope. To quantify multiple stress responses, each strain was grown in 1/4 SDAY (amended with 1/4 of each SDAY nutrient) alone (control) or supplemented with NaCl (0.4−2 M), menadione (2−8 mM), H_2_O_2_ (20−80 mM) or Congo red (0.5−3 mg/ml). The effective concentration of each chemical (EC_50_) required to suppress 50% colony growth after 6 days of incubation at 25°C was estimated by a modeling analysis of colony sizes over the gradient of concentrations [25]. All cultures were initiated by spreading 100 μl of a 10^7^ conidia/ml suspension per SDAY plate for conidiation, resuspending the conidia in SDB (10^6^ conidia/ml) for blastospore production or attaching a hyphal mass disc (5 mm diameter) to the center of each agar plate. Conidial response to each of the aforementioned chemicals was assayed by spreading the 100 μl aliquots of the 10^7^ conidia/ml suspension on to the plates with germination medium (GM: 2% sucrose and 0.5% peptone plus 1.5% agar) alone (control) or supplemented with NaCl (1.2 M), menadione (0.2 mM), H_2_O_2_ (4 mM) or Congo red (1 mg/ml), followed by estimating the relative germination rate (%) of each strain after a 24 h incubation at 25°C. Conidial thermotolerance and UV-B resistance were quantified as median lethal time (min) and dose (J/cm^2^) respectively by exposing conidial samples to 45°C of wet heat for 0−120 min and UV-B irradiation (weighted wavelength = 312 nm) at the gradient doses of 0−0.8 J/cm^2^, as described previously [25]. Conidial virulence of each strain to *Spodoptera litura* second-instar larvae was bioassayed by exposing cohorts of 30−40 larvae to a uniform spray of 1 ml of a 10^7^ conidia/ml suspension (treatment) or 0.02% Tween 80 (control) in automatic Potters Spray Tower (Burkard Scientific Ltd, Uxbridge, UK) in a standardized cabbage leaf disc system [26], generating an LT_50_ (number of days) by probit analysis of time-mortality responses. Three replicates were included in all the experiments. All phenotypic observations were subjected to one-factor (fungal strain) analysis of variance, followed by Tukey’s HSD test for the means of each phenotype between Δ*nhx1* and its control strains.

### Preparation of Protein/Phosphoprotein Samples

The WT and Δ*cdc14* were cultivated in 1/4 SDAY alone (control) or supplemented with NaCl (1 M), H_2_O_2_ (3 mM) or Congo red (0.15 mg/ml) as different stress treatments by spreading 100 μl of a 10^7^ conidia/ml suspension per cellophane-overlaid plate. After 3 days of incubation at 25°C, hyphal cultures from the control and the treatments were separately ground in liquid nitrogen, suspended in a lysis buffer [8 M urea, 1% Triton-100, 65 mM DTT (DL-dithiothreitol), 0.1% protease inhibitor Cocktail Set VI (Sigma), and 0.1% phosphatase inhibitor mixture (Roche, Mannheim, Germany)] and sonicated three times on ice under the action of a high intensity ultrasonic processor (Scientz, Shanghai, China). After 10 min of centrifugation at 20,000 ×*g* at 4°C, the supernatant was discarded and the proteins were precipitated with cold 15% TCA (trichloroacetic acid) for 2 h at –20°C, followed by washing with cold acetone three times. The precipitate was dissolved in a buffer (pH 8.0) of 8 M urea and 100 mM TEAB (tetraethylammonium bromide), and the concentration was determined with a 2-D Quant kit (GE Healthcare Bioscience, Shanghai, China). The protein solution was reduced with 10 mM DTT (dithiothreitol) for 1 h at 37°C, alkylated with 20 mM IAA (iodoacetamide) for 45 min at room temperature in darkness, and then diluted with 100 mM TEAB (tetraethylammonium bromide) until the urea concentration was below 2 M. Finally, trypsin was added to the solution at the trypsin-to-protein mass ratio of 1:50 for the first overnight digestion and of 1:100 for the second 4 h digestion. For each treatment, a protein sample of ~100 μg was digested with trypsin. The control and stress treatments of the two strains generated eight protein samples.

### iTRAQ Labeling and HPLC Fractionation

The trypsin-digested protein samples were desalted by filtration through a Strata X C18 SPE column (Phenomenex, Guangzhou, China), followed by vacuum drying. The resultant peptides were reconstituted in 0.5 M TEAB and treated with an 8-plex iTRAQ kit (Sigma) following the user’s guide. Briefly, one unit of iTRAQ^®^ reagent (an amount required to label 100 μg protein or phosphoprotein sample) was thawed and reconstituted in 24 μl of ACN (acetonitrile). Subsequently, a mixture of eight reconstituted samples was incubated for 2 h at room temperature, desalted and vacuum-dried. Finally, the sample was fractionated via high pH reverse-phase HPLC with an Agilent 300 Extend C18 column (5 μm particles, 4.6 × 250 mm). Briefly, peptides were first separated with a gradient of 2% to 60% acetonitrile in 10 mM ammonium bicarbonate (pH 10) over 80 min into 80 fractions, For the proteomic study, the peptides were combined into 14 fractions and dried by vacuum centrifuging. For the phosphoproteomic study, the peptides were combined into 4–6 fractions.

### Phosphopeptide Enrichment

Eight trypsin-digested phosphoprotein samples were mixed and incubated in a 50 μl suspension of IMAC (immobilized titanium-ion affinity chromatography) microspheres [10 mg/ml in 80% ACN and 6% TFA (trifluoroacetic acid)] by 30 min of shaking. The IMAC microspheres with enriched phosphopeptides were collected by a 5 min centrifugation at 20,000 ×*g* and washed with 100 μl solution of 50% ACN, 6% TFA and 200 mM NaCl to remove nonspecifically adsorbed peptides and then with 100 μl solution of 30% ACN and 0.1% TFA. The enriched phosphopeptides were eluted in 100 μl of 10% (v/v) NH_3_·H_2_O by 30 min of shaking, followed by a 5 min centrifugation at 20,000 ×g. The supernatant containing phosphopeptides were collected and lyophilized for LC-MS/MS analysis.

### Quantification of Peptides and Phosphopeptides by LC-MS/MS

Peptides and phosphopeptides were separately dissolved in 0.1% FA (formic acid) and then loaded onto the reverse-phase pre-column Acclaim PepMap 100 (100, 75 μm × 2 mm, 3 μm, 100 Å; Thermo Scientific). Peptide separation was performed using the reversed-phase analytical column Acclaim PepMap RSLC (50 μm × 15 mm, 2 μm, 100 Å; Thermo Scientific). The gradient of mobile phase A (0.1% FA in 98% ACN) increased from 6% to 22% over 24 min, increased from 22% to 36% in 8 min, increased to 80% in 4 min and then held at 80% for the last 4 min, all at a constant flow rate of 280 nl/min in an EASY-nLC 1000 UPLC system. Phosphopeptide separation was performed using the same analytical column. Phase A increased from 5 to 35% in 30 min and then from 35 to 80% in 10 min at a constant flow rate of 300 nl/min in the UPLC system. The resultant peptides and phosphopeptides were analyzed by means of a Q Exactive^™^ Plus Hybrid Quadrupole-Orbitrap Mass Spectrometer (Thermo Scientific).

Peptides and phosphopeptides were subjected to a NanoSpray Ionization (NSI) source, followed by tandem mass spectrometry (MS/MS) in Q Exactive^™^ Plus coupled online to the UPLC. Each of intact peptides and phosphopeptides was detected in the Orbitrap at a resolution of 70,000. Peptides and phosphopeptides were selected with 30% and 28% NCE for MS/MS analyses, respectively. Ion fragments were detected in the Orbitrap at a resolution of 17,500. A peptide data-dependent procedure alternated between one MS scan, followed by 20 and 10 MS/MS scans, to collect top 20 and 10 precursor ions of peptides and phosphopeptides above a threshold ion count of 2 × 10^4^ in the MS survey scan with 30 and 5 s dynamic exclusions, respectively, at an electrospray voltage of 2.0 kV. Automatic gain control (AGC) was used to prevent overfilling of the ion trap, and 5 × 10^4^ ions were accumulated for generation of MS/MS spectra. For MS scans, the *m/z* scan ranged from 350 to 1800, and ion charge was set in a range of 2–5.

### Database Search

The resulting MS/MS data were processed through a peptide or phosphopeptide search with the engine software MaxQuan [10]. Tandem mass spectra of peptides were searched against *B*. *bassiana* database from Uniport (10,366 sequences, Dec 2013) concatenated with a reverse decoy database. Trypsin/P was specified as a cleavage enzyme allowing for up to two missing cleavages. Mass error was set to 10 ppm for precursor ions and 0.02 Da for fragment ions. Carbamidomethyl on Cys, iTRAQ 8-plex (N-term) and iTRAQ 8-plex (K) were specified as fixed modifications, and oxidation on Met was specified as variable modifications. The false discovery rate (FDR) thresholds for protein and peptides were adjusted to < 1%, and the peptide ion score was set to > 20. For the phosphopeptide search, trypsin/P was specified as a cleavage enzyme allowing for up to four missing cleavages, four modifications per peptide and five charges at the same level of mass error. A specifically fixed modification was carbamidomethylation on Cys, while variable modifications were specified as oxidation on Met, phosphorylation on Ser, Thr and Tyr, and acetylation on protein N-terminus. FDR thresholds for protein, peptide and modification sites were specified at 1%. The minimal peptide length was set to seven residues, and site probability cutoff was set to > 75%.

All identified proteins and phosphoproteins are listed in Tables A and B in S1 File, and the default values for searching are listed in Table C in S1 File.

### Bioinformatic Analyses of Identified Proteins and Phosphoproteins

Gene Ontology (GO) analysis was performed to classify all identified proteins and phosphoproteins in three categories (cell component, molecular function and biological process) by means of the UniPort-GOA database (http://www.ebi.ac.uk/GOA/), InterProScan (http://www.ebi.ac.uk/interpro/) and GO annotation (http://geneontology.org/).

WoLF PSORT (http://wolfpsort.seq.cbrc.jp/) was used to predict if each identified protein or phosphoprotein was localized to extracellular space (extr), plasma membrane (plas), cytoplasm (cyto), nucleus (nucl), mitochondria (mito), endoplasmic reticulum (ER), peroxisome (pero), cytoskeleton (cysk) or nucleolus (nucl).

Functional category (FunCat) annotation was performed to predict intracellular roles and functions of all identified proteins and phosphoproteins in terms of the PEDANT 3 database (http://pedant.gsf.de/).

All identified proteins and phosphoproteins were subjected to analysis of eukaryotic orthologous group (KOG) by aligning their sequences with the KOG protein sequence database (http://genome.jgi.doe.gov/help/kogbrowser.jsf). The filter parameters were set to an E value < 10^−5^, sequence identity > 80%, and percent match length > 60%.

The conserved domains of each identified protein or phosphoprotein was predicted with InterProScan based on the InterPro domain database (http://www.ebi.ac.uk/interpro/).

Kyoto Encyclopedia of Genes and Genomes (KEGG) analysis (http://www.genome.jp/kegg/) was performed to annotate the functional pathways of all identified proteins or phosphoproteins.

The software Motif-x [27, 28] was used to predict motif sequences at specific positions of phospho-13-mers (six amino acids upstream and downstream of a phosphorylation site) in all identified phosphoproteins. All database protein sequences were used as the background database parameter, and other parameters were set to the default values.

Functional enrichment analysis was performed to reveal differentially expressed proteins enriched in all quantified proteins and phosphoproteins. A Fisher’s exact test method was used to gain the enriched functional terms. A differentially expressed protein or phosphoprotein was significant if *P* < 0.05.

## Results and Discussion

### Phenotypic Changes Attributed to the Deleted *cdc14*

Phenotypic changes caused by the deletion of *cdc14* in *B*. *bassiana* are summarized in Table 1. Compared with the WT, the Δ*cdc14* mutant showed a minor growth defect on SDAY, a standard medium for cultivation of entomopathogenic fungi [23], but the growth defect was worsened in MM with altered carbon/nitrogen sources. The mutant suffered from severe defects in asexual development due to a drastic reduction in aerial conidiation in a 7-day-old SDAY culture and in submerged blastospore production in a 3-day-old SDB culture. Based on more than 300 unicellular thin-wall blastospores examined under a microscope, abnormal cytokinesis occurred at a 13% frequency in Δ*cdc14* (three or more nuclei per cell) but was absent in the WT (only one or two nuclei per cell). Either colony growth or conidial germination of Δ*cdc14* versus the WT was remarkably suppressed by H_2_O_2_, menadione, Congo red and NaCl, respectively. The mutant conidia were 41% and 50% less tolerant to 45°C wet heat and UV-B irradiation, respectively, and showed a nearly double delay in lethal action against *S*. *litura* larvae under a uniform spray. All changes were restored by targeted gene complementation, hence indicating that Cdc14 acts as a critical regulator of nutritional utilization, asexual development, multiple stress responses and virulence.

**Table 1:** Effects of *cdc14* deletion on the growth, asexual development, multiple stress responses and virulence of *B*. *bassiana*.

Phenotypic measurements	Mean ± SD*		
	WT	Δ*cdc14*	Δ*cdc14/cdc14*
Colony diameter (mm) after 6-day growth on SDAY	30.0 ± 0.0 a	27.5 ± 0.5 b	30.0 ± 0.0 a
Colony diameter (mm) after 6-day growth on MM	28.4 ± 3.0 a	18.5 ± 1.4 b	28.4 ± 3.0 a
MM with glucose as sole carbon source	26.9 ± 1.5 a	14.3 ± 1.1 b	27.8 ± 0.8 a
MM with galactose as sole carbon source	26.9 ± 1.5 a	13.3 ± 0.0 b	25.9 ± 1.6 a
MM with glycerol as sole carbon source	31.5 ± 3.1 a	13.3 ± 0.0 b	31.4 ± 0.0 a
MM with acetate as sole carbon source	31.5 ± 0.2 a	15.4 ± 0.0 b	28.3 ± 0.0 a
MM with NH_4_^+^ as sole nitrogen source	26.9 ± 1.5 a	15.5 ± 1.1 b	25.4 ± 0.0 a
MM with NO_2_^−^ as sole nitrogen source	34.6 ± 0.0 a	18.9 ± 1.2 b	34.6 ± 0.0 a
Aerial conidiation level on SDAY (10^7^ conidia/cm^2^)	98.6 ± 1.6 a	3.9 ± 0.8 c	82.5 ± 6.6 b
Submerged production of 10^7^ blstospores/ml SDB	4.7 ± 0.1 a	0.5 ± 0.1c	4.5 ± 0.1 b
Frequency of multinucleate hyphae (%)	absent	13.3 ± 0.9	absent
EC_50_ for 50% suppression of colony growth by			
Oxidant H_2_O_2_ (mM)	51.1 ± 1.8 a	30.9 ± 1.9 c	45.5 ± 1.5 b
Oxidant menadione (mM)	3.5 ± 0.1 a	2.7 ± 0.2 b	3.6 ± 0.0 a
Cell-wall stressor Congo red (mg/ml)	1.3 ± 0.2 a	0.8 ± 0.0 b	1.3 ± 0.2 a
Osmotic salt NaCl (M)	1.1 ± 0.0 a	1.0 ± 0.0 b	1.1 ± 0.0 a
Relative germination (%) of conidia suppressed by			
Oxidant H_2_O_2_ (4 mM)	40.0 ± 1.0 a	21.7 ± 1.5 b	37.7 ± 1.5 a
Oxidant menadione (0.2 mM)	76.0 ± 5.3 a	23.3 ± 4.2 b	75.3 ± 3.1 a
Cell-wall stressor Congo red (0.5 mg/ml)	77.7 ± 1.5 a	58.0 ± 1.0 c	73.0 ± 2.0 b
Osmotic salt NaCl (1.2 M)	74.3 ± 2.1 a	52.0 ± 1.0 c	67.7 ± 2.5 b
LT_50_ (min) for conidial tolerance to 45°C wet heat	58.1 ± 0.6 a	34.4 ± 2.1 b	57.5 ± 1.4 b
LT_50_ (J/cm^2^) for conidial tolerance to UV-B irradiation	0.32 ± 0.02 a	0.16 ± 0.02 c	0.27 ± 0.01 b
LT_50_ (no. days) for virulence to *S*. *litura* larvae	4.3 ± 0.6 b	7.4 ± 1.1 a	4.4 ± 0.6 b

* Means followed by different lowercase letters in each line differ significantly (Tukey’s HSD, *P* < 0.05)

### Features of the Cdc14-Specific Proteome and Phosphoproteome

**Fig 1:**
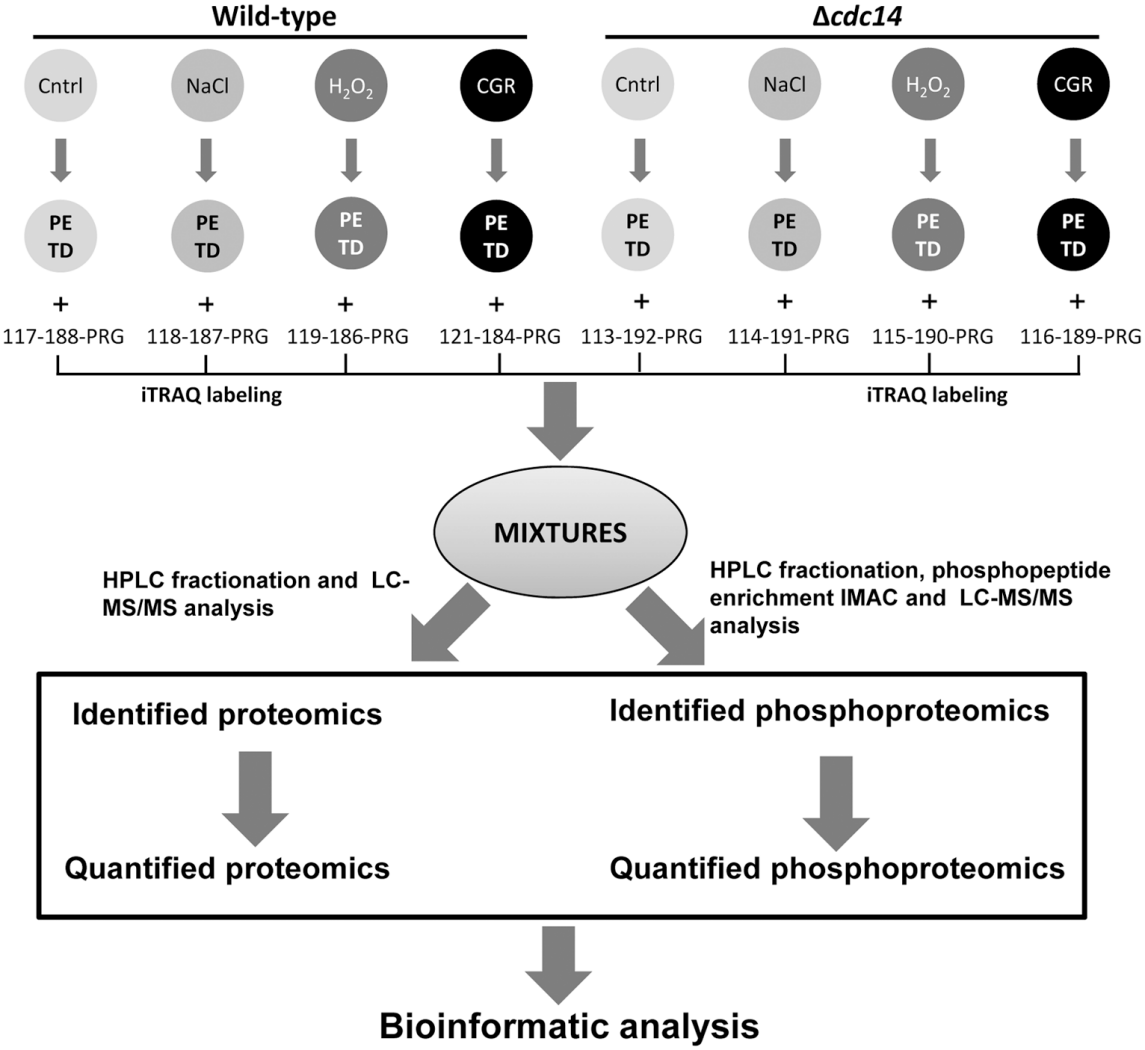
An experimental workflow for generating *B*. *bassiana* Cdc14-specific proteome and phosphoproteome.

The 8-plex iTRAQ labeling, HPLC fractionation and LC-MS/MS analysis resulted in the construction of the *B*. *bassiana* Cdc14-specific proteome and phosphoproteome (Fig 1). The *B*. *bassiana* genome consists of 10,366 genes encoding annotated and hypothetical proteins [29]. We quantified 2,251 of 3,088 proteins identified from the proteome and identified 1,048 phosphorylation sites of 479 phosphoproteins from the phosphoproteome. There were 241, 106, 63 and 69 phosphoproteins in which one, two, three and more phosphorylation sites were identified respectively.

**Fig 2:**
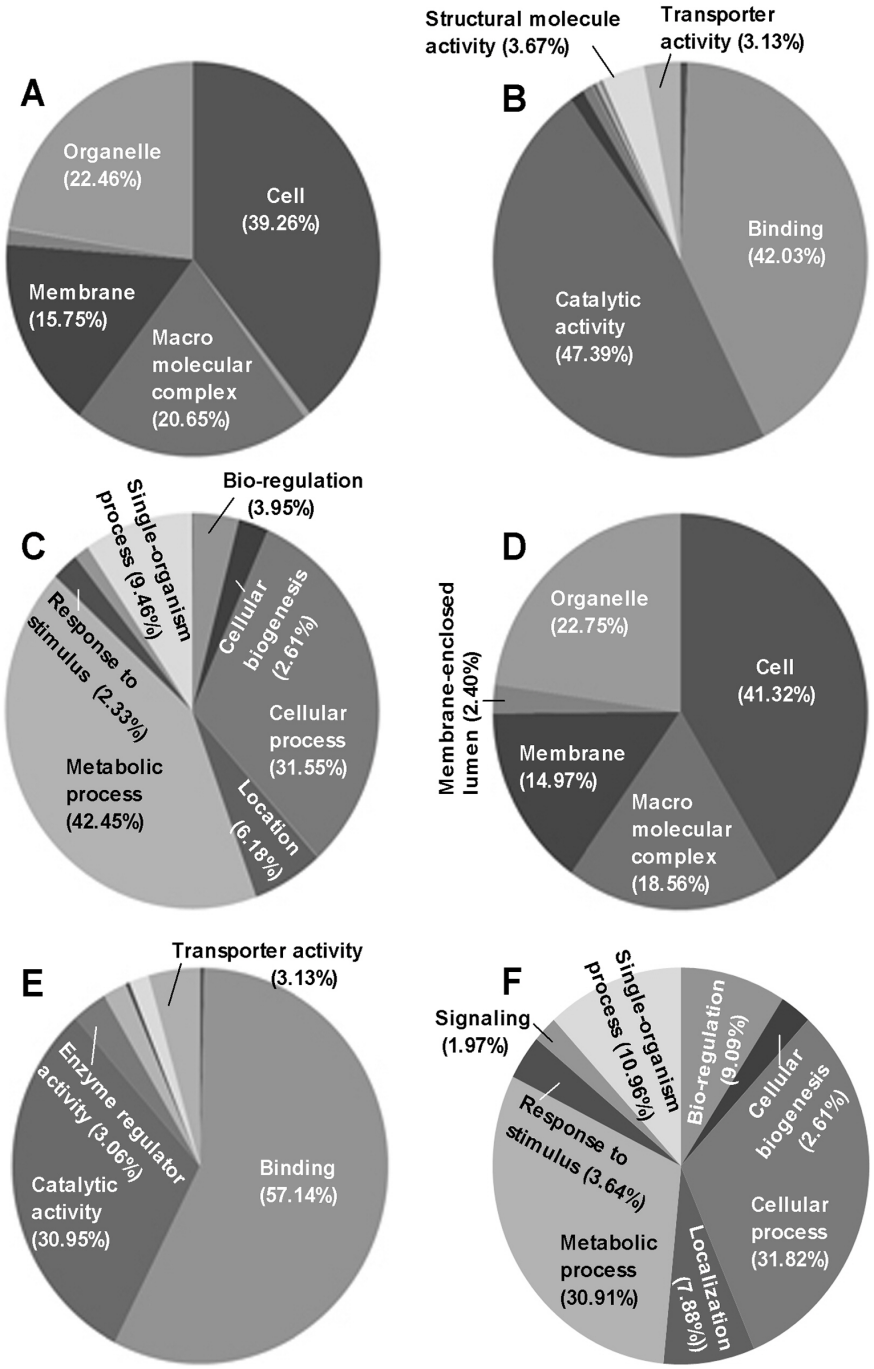
GO functional classification of quantified proteins and phosphoproteins. (**A−C**) Cell components, molecular functions and biological processes involved in the *B*. *bassiana* Cdc14-specific proteome respectively. (**D−E**) Cell components, molecular functions and biological processes involved in the *B*. *bassiana* Cdc14-specific phosphoproteome.

The GO annotation analysis of quantitative proteome and phosphoproteome resulted in the classification of proteins (Fig 2A–2C) and phosphoproteins (Fig 2D and 2E) to the categories of cell component, molecular function and biological process (Table D in S1 File). Most of them were distributed in cells, organelles, macromolecular complexes and membranes, were functionally associated mainly with binding and catalytic activities and were involved in metabolic processes, cellular processes, single-organism processes and localization. Many identified proteins were revealed as potential electron carriers, metallochaperones, and nutrient reservoirs with no phosphorylation site, suggesting that they could not be involved in the phosphorylation process. Overall, the identified proteins and phosphoproteins were diverse in each of the three categories.

**Fig 3:**
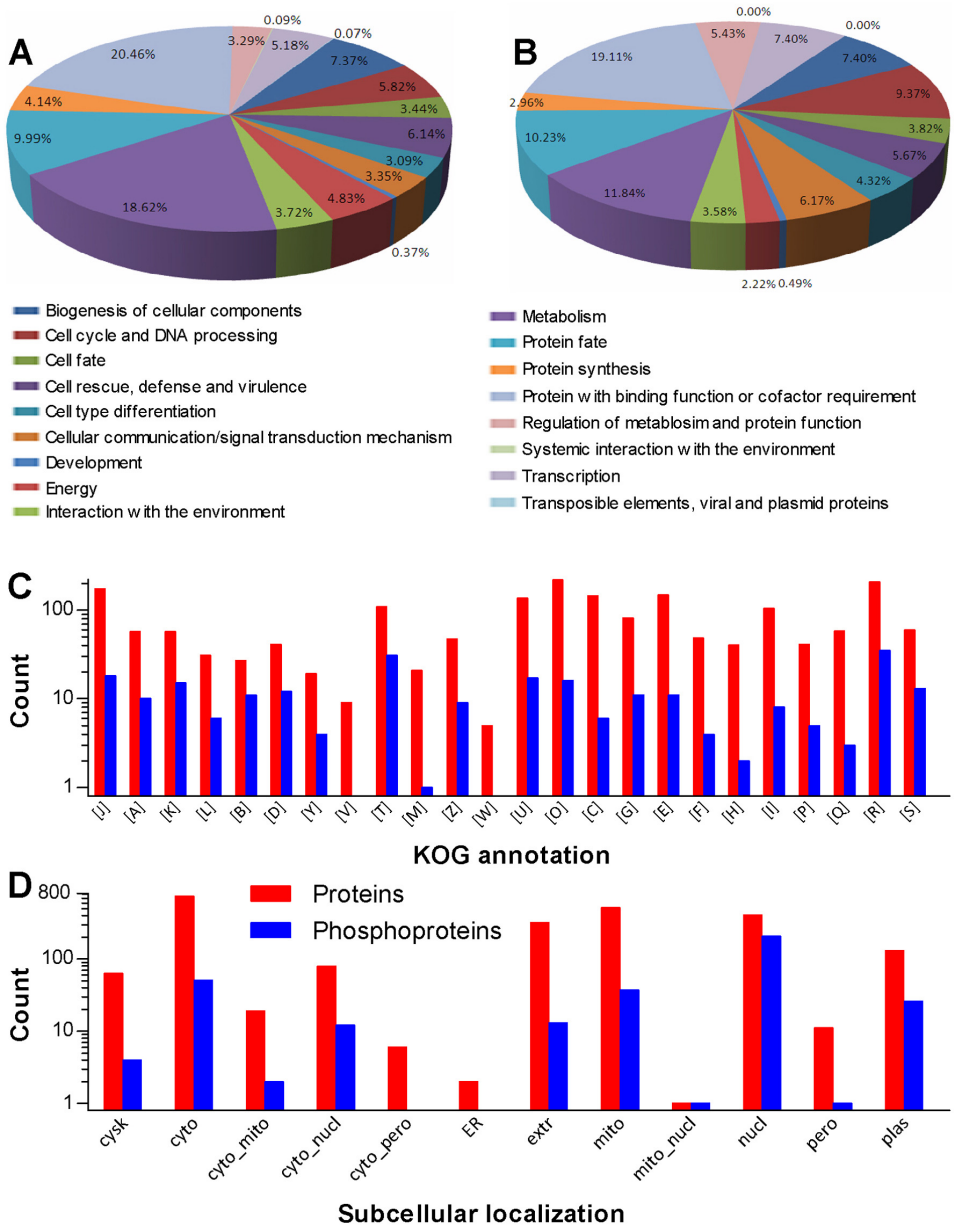
Quantification of *B*. *bassiana* Cdc14-specific proteome and phosphoproteome. (**A, B**) FunCat annotation of quantified proteins and phosphoproteins respectively. (**C**) KOG classification. (**D**) Subcellular localization predicted with WoLF PSORT.

Moreover, 1,771 proteins and 219 phosphoproteins were classified respectively to 17 and 16 function categories by FunCat analysis (Table E in S1 File). Most of them were linked to binding function or cofactor requirement, metabolism, protein fate, biogenesis of cellular components, cell rescue/defense/virulence, cell cycle and DNA processing, transcription, protein synthesis, energy, interaction with the environment, cellular communication/signal transduction mechanism, cell fate, cell type differentiation, regulation of metabolism and protein function, and development (Fig 3A and 3B). In the KOG classification, 1,649 proteins and 213 phosphoproteins were sorted into 24 groups of four levels (Fig 3C; detailed in Table F in S1 File), including function unknown groups containing 59 proteins and 13 phosphoproteins respectively. Prediction with WoLF PSORT revealed that 2,251 proteins and 352 phosphoproteins were localized to 12 and 10 cellular sites respectively, such as cytosol, mitochondria, nuclei, extracellular space and plasma membrane (Fig 3D; detailed in Table G in S1 File). Interestingly, most phosphoproteins were predicted to localize in the nuclei (205), cytosol (51), mitochondria (37) and plasma membrane (26).

**Fig 4:**
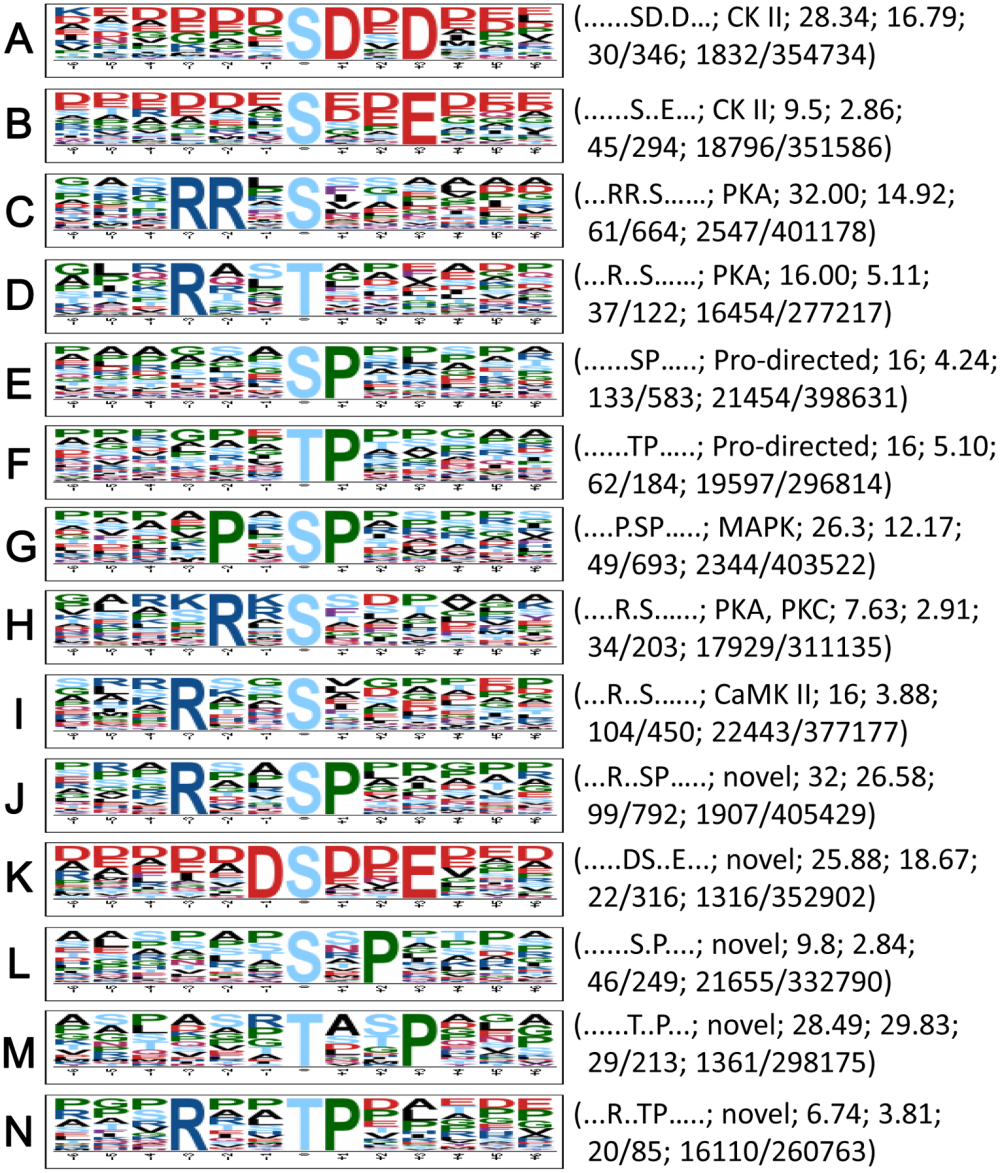
Phosphorylation motifs found in the *B*. *bassiana* Cdc14-specific phosphoproteome with Motif-x. All logos were created using WebLogo (http://weblogo.berkeley.edu). The height of each amino acid indicates the level of conservation at that position. Listed in parentheses are the sequence of each motif, potential kinase, score, fold increase, the foreground counts of matches (F_match_) and identified phosphopeptides (F_size_), and the background counts of matches (B_match_) and all the identified peptides (B_size_) in the software database, respectively. Fold increase = (F_size_/F_match_)/(B_size_/B_match_).

Qualitative phosphoproteomic analysis revealed an average of 2.2 phosphorylation sites per phosphoprotein in *B*. *bassiana* (Table H in S1 File). This estimate is similar to that in the fission yeast but smaller than those in *S*. *cerevisiae*, *Candida albicans* and *Aspergillus nidulans* [30, 31]. In eukaryotic cells, phosphorylation usually occurs, in order of preference, on serine, threonine and tyrosine. In this study, the three residues represent 78%, 21% and 1% of phosphorylation sites linked to Cdc14 in *B*. *bassiana*, respectively, corroborating previous reports. Moreover, 14 sequence motifs were predicted from all the identified phosphorylation sites with Motif-x. As illustrated in Fig 4, 623 phosphoserine peptides were matched to 10 conserved motifs, i.e., RxxSphP, PxSphP, RRxSph, SphP, RxxSph, SphDxD, DSphxxE, SphxxE, SphxP and RxSph. In these motifs, Sph and x denote a phosphorylated serine and a random amino acid residue, respectively. Four other conserved motifs were present in 148 phosphothreonine peptides, including RxxTphP, TphP, RxxTph, and TphxxP (Tph = phosphorylated threonine). However, no motif was found in any of a very few phosphotyrosine peptides.

Among the predicted motifs, nine are known to recognize the substrates of casein kinase II (CK II), protein kinase A (PKA), proline-directed kinase, MAP kinase, protein kinase C (PKC), calcium/calmodulin-dependent protein kinase II (CaMK II), and CDK [27, 28, 32, 33]. This suggests a conserved nature of phosphorylation events in eukaryotes. However, RxxSphP, DsphxxE, SphxP, RxxTphP and TphxxP) are new motifs as they were not found in the referred phosphorylation database. The phosphoproteomic analysis indicates that these highly specific phosphorylation motifs are associated with Cdc14 in *B*. *bassiana*.

### Insight into the Regulatory Role of Cdc14 in Nutritional Utilization and Asexual Development

Cdc14 has been shown to regulate positively the expression of 25 key genes involved in cytokinesis, cell division and asexual development in *B*. *bassiana* [6]. As a well-known phosphatase in *S*. *cerevisiae*, Cdc14 acts as a hub in the signaling network of 53 interaction partners, including 23 kinases and five phosphatases [5]. Revealed by the proteomic analysis in this study, 154 and 117 proteins were up- and downregulated in Δ*cdc14* versus the WT under normal culture conditions respectively. The phosphoproteomic analysis demonstrated that 86 and 85 phosphorylation sites were up- and downregulated in 67 and 58 phosphoproteins in Δ*cdc14* respectively (Table 2; detailed in Tables I and J in S1 File). These proteomic and phosphoproteomic changes imply that Cdc14 may regulate protein abundance and phosphorylation events linked to cellular communication/signal transduction, hyphal development, energy production, cell cycle and DNA replication. In enriched GO terms, KEGG pathways and protein domains, 18 proteins in the glycoside hydrolase family were downregulated in Δ*cdc14*. Most of these glycoside hydrolases are known to be involved in protein glycosylation [34], inorganic nitrogen utilization [35], carbohydrate metabolism [36–38], and glycerol synthesis [39]. Phosphorylated targets found in Δ*cdc14* included ManC (mannose-1-phosphate guanylyltransferase), NagB (glucosamine-6-phosphate isomerase), PYK1 (pyruvate kinase), GPD1 (glycerol-3-phosphate dehydrogenase), ACLY (ATP-citrate synthase) and GPH1 (starch phosphorylase). Ser-485 in the MAP kinase kinase Ste7 of the invasive growth pathway that regulates fungal growth and development [40] was also identified as a phosphorylation site of Cdc14, hinting a link of this phosphatase to the pathway.

**Table 2:** Counts of quantified proteins, phosphoproteins (P-proteins) and phosphorylation sites (P-sites) in *B*. *bassiana* Δcdc14*.

Treatment	Upregulated counts			Downregulated counts		
	Proteins	P-proteins	P-sites	Proteins	P-proteins	P-sites
Control	154	67	86	117	58	85
NaCl (1 M)	23	85	127	63	92	112
H_2_O_2_ (3 mM)	41	45	52	39	36	47
Congo red (0.15 mg/ml)	79	79	105	79	149	226

* Proteins were significantly up- or downregulated if the ratio of each protein abundance in Δ*cdc14* over that in WT was >1.5 or <0.67. Significantly up- or downregulated phosphorylation sites in phophoproteins were quantified if the phosphorylation ratio > 1.3 or < 0.77.

Moreover, Cdc14 is required for the mitotic exit network (MEN) in *S*. *cerevisiae* and septation initiation network (SIN) in the fission yeast [41–45]. In *B*. *bassiana*, abnormal cytokinesis indicated by the formation of multinucleated cells occurred at a low frequency in the absence of *cdc14*. Our analysis revealed that this phenomenon could be likely related to accumulation and phosphorylation events of many important enzymes/proteins, such as cohesin complex (SMC1, SMC3 and SCC1), DNA replication licensing factor (MCM4), casein kinase I (HRR25), casein kinase II (CSNK2), and the serine/threonine-protein phosphatase B-type regulatory subunit (RTS1). In *S*. *cerevisiae*, Cdc14 has been shown to dephosphorylate a key cyclin-dependent kinase (Cdk1) and target other cell cycle substrates, such as anaphase-promoting complex or cyclosome (APC/C) and transcriptional factor SWI5, which are crucial MEN regulators [46–48] although their phosphorylation sites have yet to be clarified. However, in *B*. *bassiana*, the phosphorylation levels of some substrates targeted by Cdc14 could be too low to be detected in the 3-day-old culture samples or other substrates could not be homologous to those in the yeast. Perhaps for this reason, the 8-plex iTRAQ labeling was unable to identify more phosphopeptides as targets of the fungal Cdc14.

### Insight into the Regulatory Role of Cdc14 in Multiple Stress Responses

Previously, transcript profiles of more than 60 signaling and effector genes involved in multiple stress-responsive pathways were largely altered by the deletion of *cdc14* in *B*. *bassiana* [6]. In this study, osmotic stress by NaCl depressed the expression of 63 proteins and 112 phosphorylation sites in 92 phosphoproteins but upregulated the expression of 23 proteins and 127 phosphorylation sites in 85 phosphoproteins in Δ*cdc14* versus the WT (Table 2; detailed in Tables K and L in S1 File). Among those, many are associated with intracellular glycerol balance by participating in the regulation of fatty acid biosynthesis, lipid biosynthesis, and pyruvate metabolism. These include fatty acid synthases FAS1 and FAS2, phospholipase PlcC, GPD1, PYK1, and glycogen synthase GYS. However, the HOG cascade (SskB, Pbs2 and Hog1) required for fungal osmoregulation [40] was not present in the listed targets of Cdc14. These imply that the regulation of osmotic response by Cdc14 could be achieved by taking part in intracellular glycerol balance rather than in the HOG pathway.

In the Δ*cdc14* response to the H_2_O_2_ stress, 39 proteins and 47 phosphorylation sites in 37 phosphoproteins were downregulated, while 41 proteins and 52 phosphorylation sites in 45 phosphoproteins were upregulated (Table 2; detailed in Tables M and N in S1 File). Those associated with the cellular response to oxidative stress included lection-like protein, sulfite oxidase SUOX, PlcC, NagB and armadillo-type fold phosphoproteins but a very few classic antioxidant enzymes, such as superoxide dismutases, catalases, peroxidases, thioredoxin proteins, and thioredoxin reductases. Exceptionally, the expression of a peroxidase (tage ID: BBA_00792) was drastically suppressed under normal and oxidative conditions. The oxidative stress also resulted in depressed phosphorylation of a peroxisomal catalase (CatP; tag ID: BBA_00792), which has been confirmed to be a major contributor to total catalase activity in *B*. *bassiana* and hence the most important regulator of the fungal response to H_2_O_2_ in the fungal catalase family [49].

Intriguingly, Δ*cdc14* had many more proteins and phosphoproteins up- or downregulated in response to the cell wall stress of Congo red than to the stress of high osmolarity or oxidation. Suppressed proteins and phosphorylation sites reached 79 and 226 (in 149 phosphoproteins), which concurred with upregulated expression of 79 proteins and 105 phosphorylation sites (in 79 phosphoproteins), respectively (Table 2; detailed in Tables O and P in S1 File). Of those, 42 putative extracellular space and plasma membrane proteins (involved in DNA topological change, gluconeogenesis and ether lipid metabolism) were downregulated by 1.5- to 5.6-fold in Δ*cdc14* versus the WT, including β-1,3-glucanosyltransferase (Gas1) and GPI-anchored surface protein (Ecm33) as positive CWI regulators in *B*. *bassiana* [14]. In the phosphoproteome, 23 phosphorylation sites in 15 phosphoproteins that are putatively localized to extracellular space and plasma membrane were significantly suppressed, including the MAP kinase kinase Mkk1 (tag ID: BBA_01095) in the CWI pathway, the MAP kinase kinase kinase SskB (tag ID: BBA_00937) in the HOG pathway, the DnaJ protein Mas5 (tag ID: BBA_03736), and the poroxidase and CatP as mentioned above. Previously, deletion of *mkk1* in *B*. *bassiana* resulted in severe defects in vegetative growth, aerial conidiation, multiple stress tolerance and host infection [50]. Mas5 has proved to be indispensible not only for osmotolerance, antioxidation and CWI but also for *in vitro* and *in vivo* life cycles in *B*. *bassiana* [51]. Particularly, the *mkk1* deletion also resulted in undetectable Hog1 phosphorylation and increased sensitivity to either cell wall perturbation or high osmolarity, suggesting a possible interplay between the CWI and HOG pathways [50]. The results from the phosphoproteomic analysis indicate that Cdc14 is involved in the interplay of the two stress-responsive pathways and hence can regulate the cellular response to not only cell wall stress but high osmolarity and oxidation in *B*. *bassiana*.

### Conclusive remarks

The 8-plex iTRAQ labeling and LC-MS/MS analyses enabled the quantification of 2,251 proteins and 593 phosphorylation sites in the Cdc14-specific proteome and phosphoproteome of *B*. *bassiana* respectively. These results provide deep insights in the regulatory role of Cdc14 in the fungal cytokinesis, nutritional utilization, vegetative growth, asexual development, multiple stress tolerance and virulence. Specifically, our phosphoproteomic analysis revealed up to 14 phosphorylation motifs as potential substrates of various protein kinases targeted by Cdc14. This highlights a hub role of Cdc14 in the signaling network of *B*. *bassiana*, as reported previously for the role of the Cdc14 homologue in *S*. *cerevisiae* [5]. The fungal phenotypes regulated by Cdc14 are largely overlapped with those of Mkk1, Mas5 and CatP (49–51), which are among the putative Cdc14 phosporylation substrates identified in this study although most of the substrates are yet to be confirmed. As an insect pathogen, *B*. *bassiana* has the broadest host spectrum among all fungal pathogens known to date [23] and hence harbors many unique proteins [29] that are perhaps required for its adaptation to most variable insect habitats. Such unique proteins could be in the lists of the identified proteins and phosphoproteins but were unable to be classified with the used software and databases. With no doubt, at least some of these unique proteins and phosphoproteins targeted by Cdc14 may play important roles in the host infection and environmental adaptation of *B*. *bassiana*, warranting future studies.

## Supporting Information

S1 File Tables A−P. (XLSX)
